# Cr_2_Te_3_ Thin Films for Integration in Magnetic Topological Insulator Heterostructures

**DOI:** 10.1038/s41598-019-47265-7

**Published:** 2019-07-25

**Authors:** D. M. Burn, L. B. Duffy, R. Fujita, S. L. Zhang, A. I. Figueroa, J. Herrero-Martin, G. van der Laan, T. Hesjedal

**Affiliations:** 1 Magnetic Spectroscopy Group, Diamond Light Source, Didcot, OX11 0DE United Kingdom; 2 Clarendon Laboratory, Department of Physics, University of Oxford, Parks Road, Oxford, OX1 3PU United Kingdom; 3 ISIS, Rutherford Appleton Laboratory, Science and Technology Facilities Council, Oxon, OX11 0QX United Kingdom; 4 Catalan Institute of Nanoscience and Nanotechnology (ICN2), CSIC, Campus UAB, Barcelona, 08193 Spain; 5 CELLS-Divisió Experiments, ALBA Synchrotron Light Source, E-08290 Cerdanyola del Vallès, Barcelona, Catalonia Spain

**Keywords:** Topological insulators, Ferromagnetism

## Abstract

Chromium telluride compounds are promising ferromagnets for proximity coupling to magnetic topological insulators (MTIs) of the Cr-doped (Bi,Sb)_2_(Se,Te)_3_ class of materials as they share the same elements, thus simplifying thin film growth, as well as due to their compatible crystal structure. Recently, it has been demonstrated that high quality (001)-oriented Cr_2_Te_3_ thin films with perpendicular magnetic anisotropy can be grown on *c*-plane sapphire substrate. Here, we present a magnetic and soft x-ray absorption spectroscopy study of the chemical and magnetic properties of Cr_2_Te_3_ thin films. X-ray magnetic circular dichroism (XMCD) measured at the Cr *L*_2,3_ edges gives information about the local electronic and magnetic structure of the Cr ions. We further demonstrate the overgrowth of Cr_2_Te_3_ (001) thin films by high-quality Cr-doped Sb_2_Te_3_ films. The magnetic properties of the layers have been characterized and our results provide a starting point for refining the physical models of the complex magnetic ordering in Cr_2_Te_3_ thin films, and their integration into advanced MTI heterostructures for quantum device applications.

## Introduction

Ferromagnetic materials, which are compatible with semiconductors in terms of their crystal structure and deposition conditions, have been intensely studied for applications in spintronics^1^. Apart from traditional semiconductor spintronics, the combination of magnetic materials and topological insulators (TIs) has been a promising route for observing new quantum effects at more easily accessible temperatures^2,3^. Transition metal doped magnetic TIs (MTIs) grown by molecular beam epitaxy (MBE), such as Cr-doped (Bi,Sb)_2_Te_3_, have been pivotal for observing the quantum anomalous Hall effect^4^.

The lattice-matched *antiferromagnet* CrSb and ferromagnetic Cr_2_Ge_2_Te_6_ have been shown to be ideal for the combination with Cr-doped (Bi,Sb)_2_Te_3_ MTI layers in heterostructures and superlattices, allowing for the engineering of their electronic and magnetic properties^3,5^. A key advantage of CrSb is the fact that no additional elements are needed for their MBE growth. On the other hand, chromium telluride compounds are promising *ferromagnets* for the integration with Cr-doped (Bi,Sb)_2_Te_3_ as they also share the same elements and due to their compatible crystal structure. Recently, the epitaxial growth of high quality (001)-oriented Cr_2_Te_3_ thin films with perpendicular magnetic anisotropy has been demonstrated on *c*-plane sapphire and Si(111) substrates using MBE^6^. A systematic study of the structural and magnetic properties of Cr_2_Te_3_ films on CdTe(001) substrates showed that the epitaxial relationship of the film with the substrate depends on the Cr:Te flux ratio during growth^7^. Cr_2_Te_3_ has also been grown in the form of nanorods using a high-temperature organic-solution-phase method^8^.

**Figure 1:**
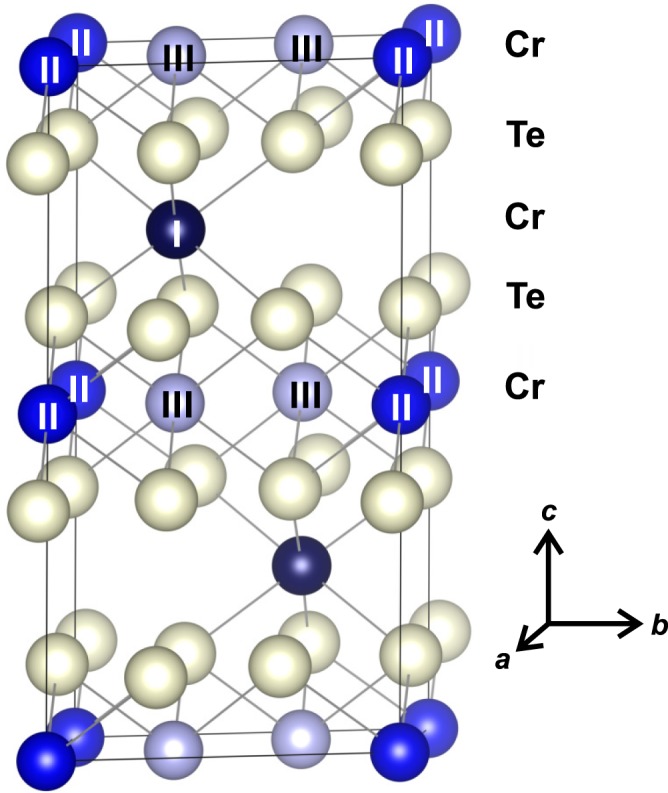
Crystal structure of Cr_2_Te_3_. The unit cell is characterized by an alternating sequence of Cr and Te layers, with Cr vacancies occurring in every other Cr layer. The distinct Cr atoms, Cr _I_ in the vacancy layer, as well as Cr _II_ and Cr _III_ in the fully occupied layer, are labeled. Note that the roman numbers in the subscripts refer to the different sites and not to the valencies of Cr.

The chromium tellurides with metal-deficient NiAs-type crystal structures, Cr_1−*δ*_Te, have in common a (distorted) hexagonal close packing of Te atoms, with Cr atoms in octahedral interstices. The crystal structure of Cr_2_Te_3_ (see Fig. 1) can be described in the space group $P\bar{3}1c$ or ${D}_{3d}^{2}$ (#163 in the International Tables of Crystallography), with the atoms on the special positions^9^. The lattice parameters are *a* = 6.814 Å and *c* = 12.073 Å^10^. The unit cell appears layered and is characterized by an alternating sequence of Cr and Te layers, while Cr vacancies occur in every second metal layer. Consequently, there are distinct Cr positions as shown in Fig. 1: Cr_I_ atoms have no direct neighbors in the *ab* plane, but are accompanied by Cr_III_ atoms in the neighboring Cr layers above and below. Cr_II_ atoms, on the other hand, are the neighbor of Cr_III_ in the filled Cr layers, however, not accompanied by any Cr atoms along the *c*-axis in the adjacent Cr layers. While the Cr sites at the layers containing Cr_II_ and Cr_III_ are completely filled, the other layers are only partially occupied by Cr_I_ atoms with vacancies. From the crystal structure in Fig. 1, the exchange interaction between Cr_II_ and Cr_III_ is expected to be large as they are close together. On the other hand, the interaction of Cr_I_ with the more distant Cr_II_ and Cr_III_ ions is expected to be relatively weak because its nearest neighbor is Te.

In the form of thin films, the most relevant compounds are the half-metal CrTe (*δ* = 0, zincblende), Cr_3_Te_4_ (*δ* = 0.25, monoclinic), and Cr_2_Te_3_ (*δ* = 0.33, trigonal), which are all metallic ferromagnets with ordering temperatures ranging from 100–340K^9–12^. Cr_2_Te_3_ is of particular interest in the context of MBE-grown thin films with a reported *T*_C_ ≈ 183K. Neutron diffraction showed that the magnetic moments of Cr in the fully occupied layers are ferromagnetically aligned and have an average value of 2.6 *μ*_B_/atom, while the Cr atoms in the partially filled layers are assumed to have only a small moment^10^. The interlayer coupling along the *c*-axis is weak. For Cr_2_Te_3_, the electronic band-structure calculations show that Cr 3*d*–Te 5*p* covalency and Cr $3{d}_{{z}^{2}}$–Cr $3{d}_{{z}^{2}}$ overlap along the *c*-axis are the most important interactions. The magnetic polarization of Te is antiparallel to the Cr moment with calculated average values of *μ*_Cr_ = 3.30 *μ*_B_/Cr and *μ*_Te_ = −0.18 *μ*_B_/Te, respectively^9^.

However, the relatively complicated magnetic structure of Cr_2_Te_3_ is not fully understood yet, in parts owing to the various ways the spins of the three distinct Cr sites can couple^13^. In fact, from electron spin resonance measurements of bulk crystals, it was claimed that the true Curie temperature is 335K, and that the generally reported *T*_C_ of 198K is only another type of magnetic transition point^14^.

Here, we present a magnetic and soft x-ray absorption spectroscopy study of the chemical and magnetic properties of Cr_2_Te_3_ thin films. We further demonstrate the successful growth of Cr_2_Te_3_/Cr:Sb_2_Te_3_ heterostructures, opening the door for advanced heterostructures combining proximity coupling to ferromagnetic layers.

## Results and Discussion

### Structural properties

**Figure 2:**
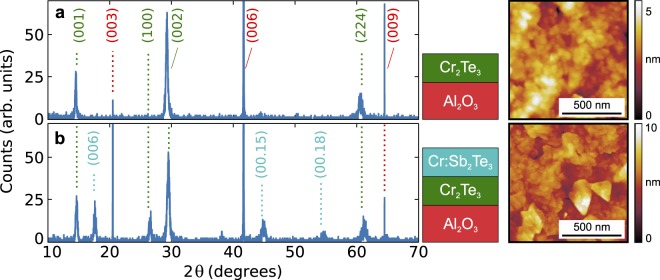
X-ray diffraction characterization. (**a**), Cr_2_Te_3_, and (**b**), Cr:Sb_2_Te_3_/Cr_2_Te_3_ thin film samples on *c*-plane sapphire. The peaks from the Al_2_O_3_ substrate, and the Cr_2_Te_3_ and Cr:Sb_2_Te_3_ thin films, have been indexed (shown in red, green, and cyan, respectively). The small peak at 2*θ* = 38° in (**b**) is originating from the Al sample holder. On the right-hand side, 1 × 1 *μ*m^2^ AFM scans show the morphology of the Cr_2_Te_3_ and Cr:Sb_2_Te_3_ surfaces, respectively, with the latter showing the characteristic triangular strictures.

The structural quality of the samples was characterized using XRD. The results for both single-layer Cr_2_Te_3_ and Cr_2_Te_3_/Cr:Sb_2_Te_3_ are shown in Fig. 2. The spectrum for single-layer Cr_2_Te_3_ (Fig. 2a) is characterized by film and sapphire substrate peaks, whereas the Cr_2_Te_3_/Cr:Sb_2_Te_3_ spectrum (Fig. 2b) additionally shows Sb_2_Te_3_ related peaks. These are shifted to higher angles with respect to undoped Sb_2_Te_3_ as Cr doping reduces the lattice spacing^15–17^.

### Magnetic behavior

**Figure 3:**
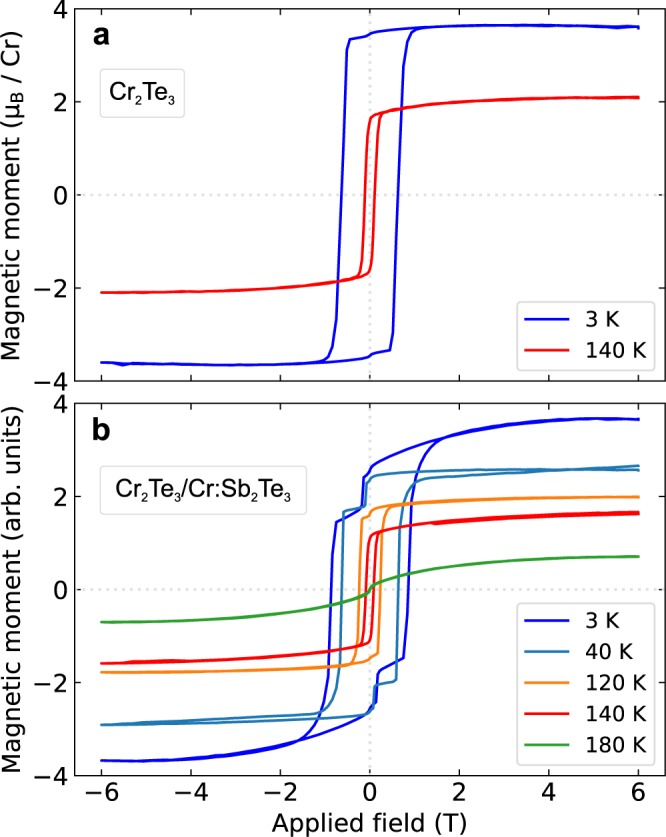
Magnetization as a function of applied out-of-plane field. (**a**), Cr_2_Te_3_, and (**b**), Cr_2_Te_3_/Cr:Sb_2_Te_3_. The measurements were performed at various temperatures as indicated. In (**a**) the average moment per Cr is shown, whilst in (**b**) the units are arbitrary as both layers contain Cr in different environments.

The magnetic characterization of the films was performed through SQUID measurements as a function of both applied magnetic field and temperature. Figure 3a,b show hysteresis loops of the magnetization as a function of applied magnetic field for Cr_2_Te_3_ and Cr_2_Te_3_/Cr:Sb_2_Te_3_, respectively.

The Cr_2_Te_3_ films show square hysteresis loops with the magnetization reversing to its saturated state during a sharp transition over a narrow field range (Fig. 3a). At 3K, the magnetization is saturated at 3.6 *μ*_B_/Cr and a coercive field of 600 mT is required to reverse the magnetization At the higher temperature of 140K, the coercivity reduces to 180 mT and is accompanied by a decrease in the saturation magnetization as the temperature approaches *T*_C_.

The Cr_2_Te_3_/Cr:Sb_2_Te_3_ bilayers show a more complex magnetization reversal with additional features associated with the MTI layer). At 140K, the magnetization reversals in Fig. 3a,b look similar with a sharp reversal at a low coercive field of 180 mT. This suggests the magnetic response is dominated by the Cr_2_Te_3_ layer at this temperature.

When the temperature is reduced, the magnetization reversal associated with the Cr_2_Te_3_ layer occurs at an increased coercive field, consistent with the measurements on single-layer Cr_2_Te_3_. However, a second magnetization reversal process arises with a low coercivity. This is attributed to a magnetic component in the Cr:Sb_2_Te_3_ film with a lower Curie temperature.

**Figure 4:**
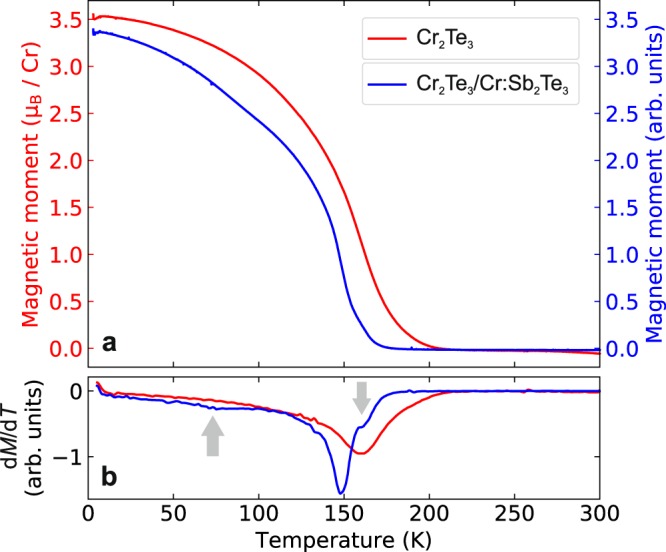
Temperature dependence of the magnetization. (**a**), *M*(*T*), and (**b**), its derivative, measured with SQUID for Cr_2_Te_3_ and Cr_2_Te_3_/Cr:Sb_2_Te_3_ samples. The measurements were performed in a field of 20 mT with increasing temperature after preparation in a 6T field-cooled state. The moment for single-layer Cr_2_Te_3_ is shown in *μ*_B_/Cr, while for the bilayer sample, the units are arbitrary due to the mixed Cr environments. Kinks at ~70K and 160K in the Cr_2_Te_3_/Cr:Sb_2_Te_3_ derivative plot are indicated by arrows.

Further SQUID magnetometry measurements were carried out to investigate the change in magnetic behavior at different temperatures. Figure 4a,b show both the magnetization and its derivative with respect to temperature as a function of increasing temperature after field-cooling in a field of 6T, respectively. At low temperatures after field cooling, Cr_2_Te_3_ has a large moment which decreases with increasing temperature. The Curie temperature, as defined as the peak in the derivative, is ~16K. The Cr_2_Te_3_/Cr:Sb_2_Te_3_ sample shows a similar variation of the magnetization with temperature, with a similar *T*_C_ of ~15K. However, there are additional subtle features, such as kinks at 70K and 160K (see Fig. 4).

The variation in magnetization as a function of temperature from the field cooled measurements is consistent with the saturation magnetization found in Fig. 3. Furthermore, the feature at 70K indicates the point at which the two-step magnetization reversal occurs in Fig. 3b.

### X-ray magnetic circular dichroism

**Figure 5:**
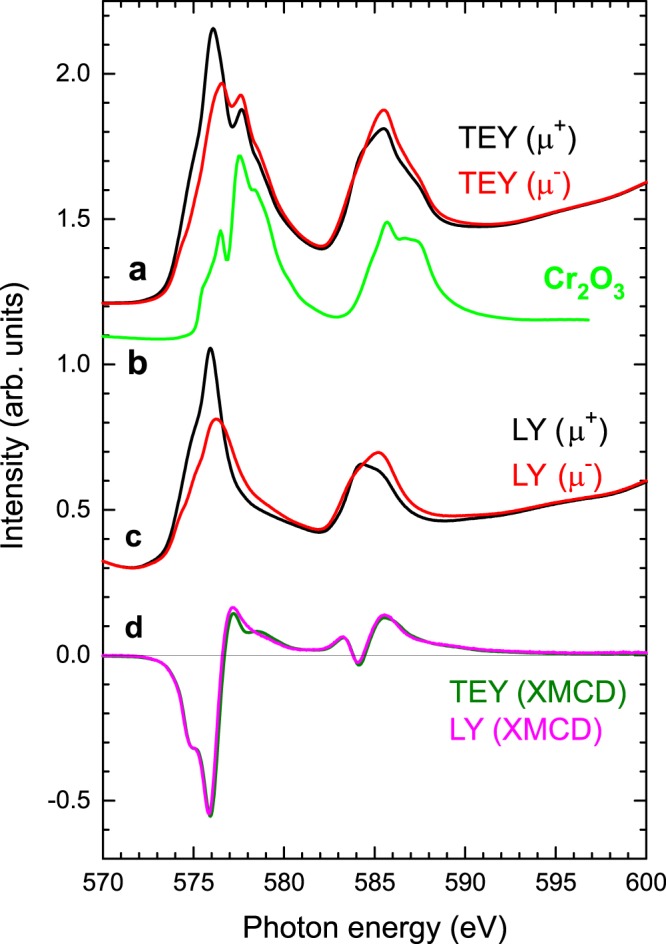
Experimental Cr *L*_2,3_ XAS and XMCD of a surface-oxidized, ~100-nm-thick film of Cr_2_Te_3_. Measurement carried out in a 6T field at 3K with 30° grazing incidence angle of the x-rays. (**a**), The total-electron yield (TEY) spectra for *μ*^+^ and *μ*^−^ (field parallel and antiparallel to incident photon helicity, respectively). (**b**), For comparison, the Cr_2_O_3_ XAS, which quite well matches the second peak in the TEY of Cr_2_Te_3_, which demonstrates that the film is oxidized at the surface. (**c**), The luminescence yield (LY) spectra for *μ*^+^ and *μ*^−^, which show no oxidation. (**d**), The XMCD measured in TEY and LY. Spectra are normalized to the size of the maximum XMCD signal.

Before showing the results from a clean sample, we first present the results from a surface-oxidized sample, in order to show the effect of oxidation on the spectra. Figure 5a shows the Cr *L*_2,3_ edge of a ~100-nm-thick Cr_2_Te_3_ film measured with surface-sensitive TEY detection. Comparison with a Cr_2_O_3_ reference sample (Fig. 5b) clearly indicates that the Cr_2_Te_3_ film surface is oxidized (due to aging). Oxidation leads to an additional Cr^3+^ peak. The photon energy of this peak is 1.5 eV higher than the photon energy of the proper Cr peak.

Figure 5c shows the spectra across the Cr *L*_2,3_ edges in LY mode. These spectra represent an average over the entire thin film heterostructure. The background variations in the LY arise from the x-ray absorption near edge structure (XANES) O *K* edge above ~543 eV from the substrate sapphire (Al_2_O_3_)^18^. Furthermore, the Cr *L*_2,3_ edges coincide with Te *M*_4,5_ edges, resulting in a sloping background of the XAS. The integrated intensity ratio of Te *M*_4,5_/Cr *L*_2,3_ XAS is estimated to be ~7.5%^19^, meaning that the Te contribution is small.

Figure 5d shows the XMCD simultaneously detected with TEY and LY. The spectra have been normalized to the maximum of the Cr *L*_3_ XMCD signal. As expected, the overall XMCD intensity of the *L*_3_ peak is negative and that of the *L*_2_ peak positive, resulting from the spin-orbit interaction of the 2*p* core levels^20^. Although antiferromagnetic Cr_2_O_3_ has zero XMCD signal, the surface-sensitive TEY of Cr_2_Te_3_ XMCD shows a more detailed structure at the high end of the Cr *L*_3_ edge (577–580 eV). These differences in the XMCD are small in relation to those in the XAS. This means that the oxidized surface hardly contributes to the magnetic signal, and it is likely to be mainly antiferromagnetic as in the case of Cr_2_O_3_.

**Figure 6:**
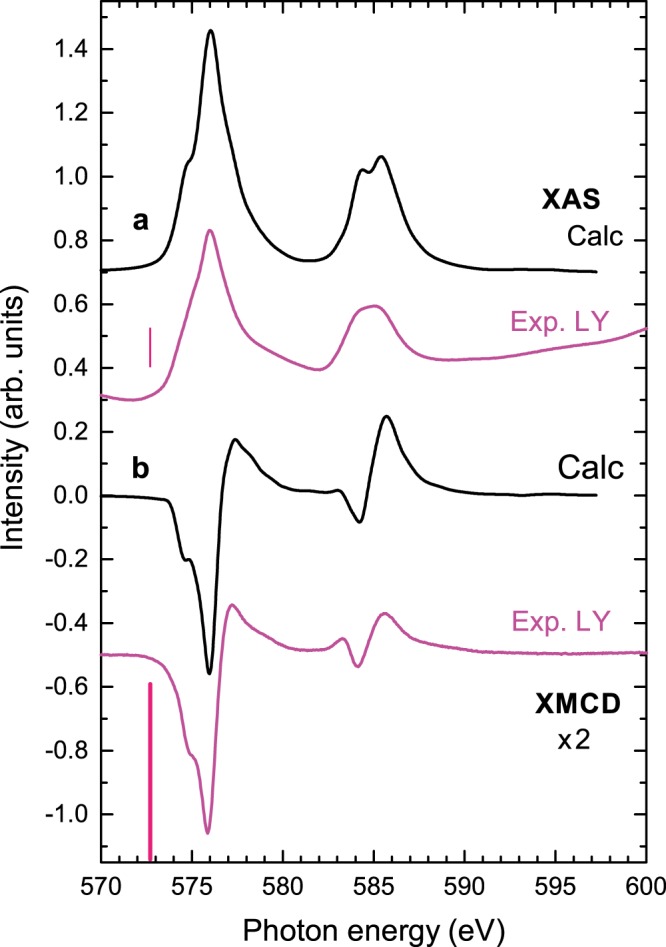
Cr *L*_2,3_ spectra of a clean, ~100-nm-thick film of Cr_2_Te_3_. Measurement carried out in a 6T field at 3K with 30° grazing incidence angle of the x-rays. (**a**), The XAS (average of *μ*^+^ and *μ*^−^) obtained from multiplet calculations and as measured in LY. (**b**), The XMCD (*μ*^+^ − *μ*^−^) as calculated and as measured in LY. The red lines at 572.7 eV mark the location of the small Te *M*_5_ peak expected in the LY data, which is not clearly evident here. Spectra are normalized to the size of the maximum XMCD signal.

Figure 6 shows the XAS and XMCD of a non-oxidized Cr_2_Te_3_ thin film. In the XMCD a small negative Te *M*_5_ signal is expected at ~572.7 eV as we earlier reported for Cr:Sb_2_Te_3_^21^. In Fig. 6, this is however untraceable in the coinciding Cr multiplet structure. The azimuthal quantum numbers of the orbitals in these electric-dipole transitions are 3*d* → 5*p* for Te and 2*p* → 3*d* for Cr, i.e., opposite. Consequently the Te and Cr moments are aligned antiparallel^17,20^ as expected from band structure calculations^9^.

For comparison, theoretical calculations for the Cr *L*_2,3_ XAS and XMCD are also shown in Fig. 6, and are discussed below.

### Multiplet calculations

We employed atomic multiplet theory to calculate the electric-dipole transitions from 3*d*^*n*^ to 2*p*^5^3*d*^*n*+1^ ^22,23^. For this purpose, the wave functions of the configurations of the initial and the final state are obtained in intermediate coupling using Cowan’s atomic Hartree-Fock (HF) code with relativistic corrections^24,25^. The 2*p*-3*d* and 3*d*-3*d* Coulomb and exchange interactions are included in the atomic electrostatic interactions. To account for the intra-atomic screening, their atomic HF value is reduced by 30%^22^. By mixing the 3*d*^*n*^ with $3{d}^{n+1}\underline{L}$ configurations with a transfer integral, *V*, hybridization effects are included. $\underline{L}$ represents a hole in the overlapping Te 5*p* orbitals.

The local ground state of Cr is taken as a coherent mixture of *ψ*(3*d*^3^) and $\psi (3{d}^{4}\underline{L})$ states. Similar to the calculation by Yaji *et al*.^19^, we obtain a mixed ground state of 54% Cr^3+^ *d*^3^ and 46% Cr^2+^ ${d}^{4}\underline{L}$ using the parameters ${{\rm{\Delta }}}_{i}\equiv E(3{d}^{4}\underline{L})-E(3{d}^{3})$ = 0 eV. As the presence of a core hole reduces the energy of the 3*d* states, Δ_*f*_ defined as $E(2{p}^{5}3{d}^{4}\underline{L})-E(2{p}^{5}3{d}^{3})$ is −1 eV, the octahedral crystal field of 10*Dq* is 1.5 eV, and the mixing transfer integral *V* is 1.5 eV. To account for intrinsic lifetime broadening and instrumental broadening, the calculated Cr *L*_3_ (*L*_2_) line spectra are broadened by a Lorentzian and a Gaussian function, respectively. The Lorentzian has a half-width at half-maximum of Γ = 0.3 eV (0.4 eV) and the Gaussian a standard deviation of *σ* = 0.15 eV. The calculated XAS and XMCD are shown in Fig. 6.

The obtained covalent character of Cr_2_Te_3_ can be ascribed to the hybridization between the Cr *d*(*e*_*g*_) and Te 5*p* bands, which are located just above and below the Fermi level, respectively.

The Cr state for the covalent compound Cr_2_Te_3_ (54% *d*^3^ and 46% *d*^4^ character in the wave function) resembles that of (V,Cr)_*x*_Sb_2−*x*_Te_3_ (ref.^26^), but strongly differs from that of Cr_*x*_Sb_2−*x*_Te_3_ (ref.^17^) and Cr_*x*_Bi_2−*x*_Se_3_ (refs^15,27^). In the latter, Sb and Bi are substitutionally replaced by Cr. In this case, Cr is nominally divalent and has 30% *d*^3^ and 70% *d*^4^ character.

Using the sum rules, the orbital and spin magnetic moments are obtained from the integrated *L*_2,3_ XAS and XMCD intensities^28,29^. For obtaining the spin moment from the sum rules, a correction factor has to be taken into account to include the *jj* mixing between the 2*p*_3/2_ and 2*p*_1/2_ manifolds^15^. This correction can result in a substantial error bar for Cr. Therefore, we determined instead the spin and orbital moments from the calculated ground state, which gives *μ*_spin_ ≈ 3.5 *μ*_B_/Cr (i.e., high spin moment) and *μ*_orb_ ≈ −0.1 *μ*_B_/Cr (i.e., opposite sign).

### XMCD magnetization loops

**Figure 7:**
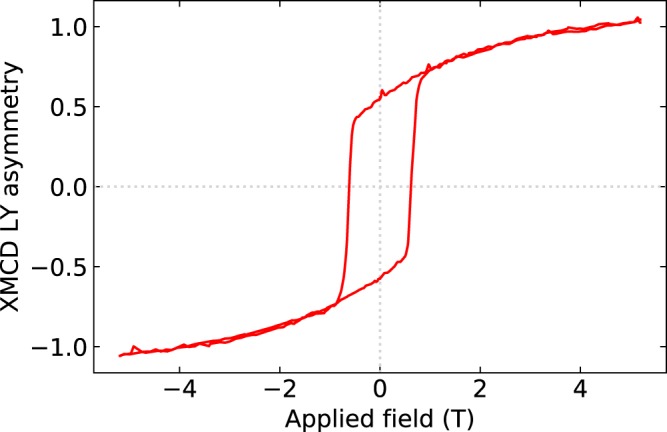
Asymmetry in the luminescence yield of Cr_2_Te_3_ measured at the Cr *L*_3_ edge. The data represent the element-specific magnetization for Cr as a function of the applied magnetic field component out-of-plane of the sample. Measurements are performed at 3K with an x-ray incidence angle of 30°.

The XMCD furthermore provides a means to explore the element-specific hysteresis loops within our multilayer samples^20^. Figure 7 shows the Cr magnetization as a function of applied field at 3K with the photon energy tuned to the maximum of the XMCD at the Cr *L*_3_ edge. Both the loop shape and coercivity of the Cr XMCD hysteresis loop are consistent with the SQUID measurements of the bulk sample shown in Fig. 3a. This shows the magnetization of the Cr dominates the magnetization in the bulk sample.

We note also that the XMCD hysteresis loop is present on a curved background. This is likely to result from the XMCD measurements being performed at an angle of 30°. Therefore, there is a component of the in-plane loop which also includes a continual rotation of the moments into the field direction at higher fields.

**Figure 8:**
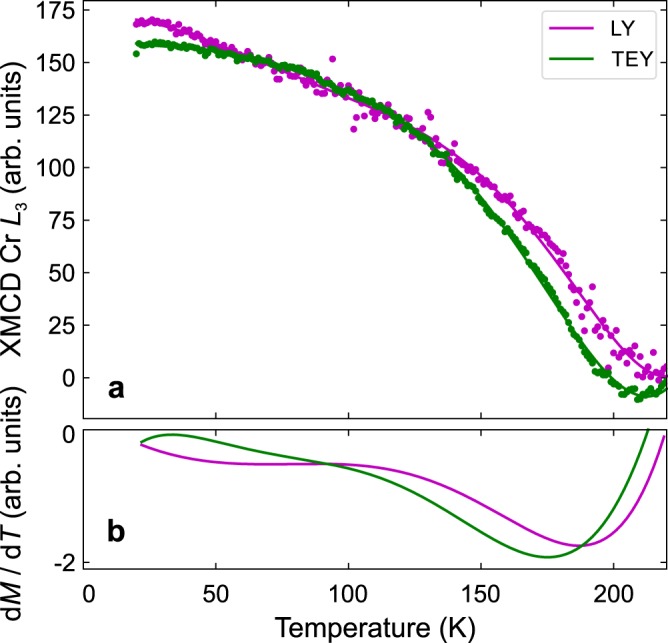
Element-specific magnetization of Cr_2_Te_3_ measured at the Cr *L*_3_ edge. (**a**), Luminescence yield (LY) and total-electron yield (TEY) as a function of temperature, and (**b**), their derivatives. The sample was initially field-cooled in 6T before measuring from 10 to 250K with a constant applied field of 20 mT with an x-ray incidence angle of 30°.

Measurements of the Cr magnetization as a function of temperature were also performed on the Cr_2_Te_3_ film and are shown, along with the derivative d*M*/d*T*, in Fig. 8a,b. Here, the LY and TEY signals are collected simultaneously. The solid lines present a polynomial fit to the data. The behavior of the magnetization in Cr shows the same trend as observed in the SQUID measurements (Fig. 4) except for the *T*_C_ measured by x-rays appears slightly higher. This is likely to be due to temperature lag in the instrumentation. The *T*_C_, as obtained from d*M*/d*T*, for the TEY signal is ~8K lower than for the LY signal, possibly pointing towards an enhanced magnetic ordering temperature at the surface compared to the bulk.

## Conclusions

We have studied the structural and magnetic properties of MBE-grown Cr_2_Te_3_ films on *c*-plane sapphire, in comparison with Cr_2_Te_3_/Cr:Sb_2_Te_3_ bilayer samples. The field and temperature dependence of the magnetization has been explored through SQUID magnetometry where a ferromagnetic response in Cr_2_Te_3_ arises below *T*_*C*_ = 150K with a coercivity increasing with decreasing temperature. In bilayer samples with Cr:Sb_2_Te_3_, a second transition temperature at ~7K shows an onset of a two-step magnetization reversal process with the reversal of the MTI layer at a lower field. Using soft x-ray absorption spectroscopy we determined the chemical and magnetic properties of Cr_2_Te_3_ thin films. The field and temperature dependent Cr XMCD matches that of the bulk magnetometry measurements and confirms the full moment originates on the Cr sites. Comparison of the Cr *L*_2,3_ spectral shapes with multiplet calculations gives a hybridized Cr state of 54% Cr^3+^ 3*d*^3^ and 46% Cr^2+^ 3*d*^4^ character in an octahedral crystal-field symmetry with spin and orbital moments of *μ*_spin_ ≈ 3.5 *μ*_B_/Cr and *μ*_orb_ ≈ −0.1 *μ*_B_/Cr.

## Methods

### Structural characterization

The thin film samples were grown by MBE on 1/4-2” diameter, *c*-plane sapphire wafers. This study focuses on Cr_2_Te_3_ thin films (typical thickness 100 nm) which were grown in (001) orientation at a substrate temperature of 400 °C and at a rate of ~1.7 nm/min on Al_2_O_3_(0001). Some of the films were further overgrown with ~3 nm thick Cr-doped Sb_2_Te_3_ at a substrate temperature of 250 °C, as described in detail in ref.^30^. Cr_*x*_Sb_2−*x*_Te_3_ is an MTI with out-of-plane magnetic anisotropy and a Cr concentration-dependent transition temperature of up to 125K. The nominal Cr concentration was *x* = 0.25. X-ray diffraction (XRD) was carried out on a Bruker D8 diffractometer using incident Cu-*Kα*_1_ radiation. The XRD measurements provide an indication of the Cr-Te phase and the crystalline quality of the films. Atomic force microscopy (AFM) was used to characterize the surface morphology of the samples.

### Magnetometry

Magnetic characterization of the samples was performed through SQUID magnetometry with a field applied out-of-plane, along the *c*-axis of the sample. After field-cooling in a field of 6T, the magnetization as a function of increasing temperature from 3 to 300K was measured in a magnetic field of 20 mT. The magnetization as a function of applied field was also measured at various temperatures above and below *T*_C_.

### X-ray absorption spectroscopy and x-ray magnetic circular dichroism

**Figure 9:**
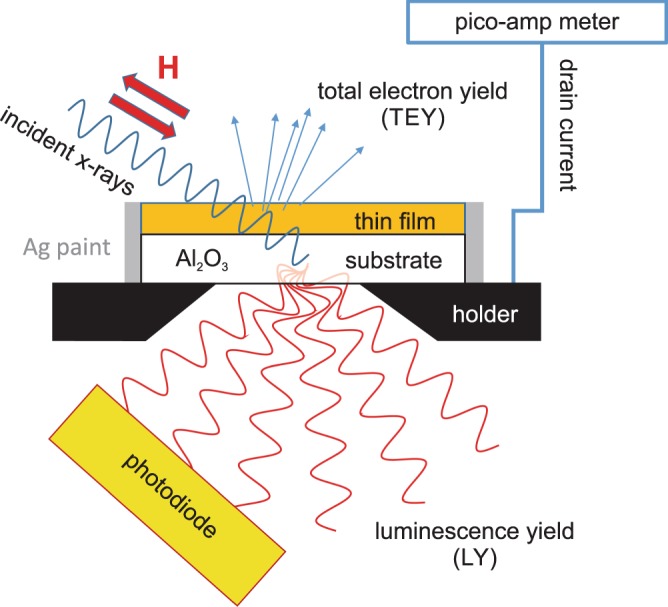
X-ray absorption spectroscopy and x-ray magnetic circular dichroism measurements. Schematic setup for simultaneous detection of total-electron yield (TEY) and luminescence yield (LY) in the vacuum chamber of the magnet. In TEY, the drain current (compensation current) is a measure for the amount of emitted electron due to x-ray absorption. TEY is surface-sensitive. In LY, which probes the entire thin-film sample, the transmitted x-rays that are not absorbed in the sample stack give rise to x-ray excited optical luminescence in the sapphire substrate. The emitted optical photons exit through a hole in the back of the sample holder and are detected by a photodiode.

X-ray absorption spectroscopy (XAS) and x-ray magnetic circular dichroism (XMCD) measurements were performed on beamline 29 (BOREAS) at the ALBA synchrotron in Barcelona, Spain^31^. XAS was simultaneously measured at the Cr *L*_2,3_ edge in total-electron-yield (TEY) mode and luminescence-yield (LY) mode (see Fig. 9 for a schematic of the measurement modes). The Cr *L*_2,3_ photon energy region coincides with that of the Te *M*_4,5_ edges. TEY provides surface-sensitive measurements with a probing depth of 3–5 nm^20^. On the other hand, luminescence-yield (LY) mode probes the entire thin film sample. In the latter mode, the transmitted x-rays that are not absorbed in the sample stack give rise to x-ray excited optical luminescence in the sapphire substrate. The emitted optical photons exit through a hole in the back of the sample holder and are detected by a photodiode^21^. The XAS spectra were calculated by taking the negative logarithm of the LY intensity after normalizing it by the incident beam intensity.

The XMCD was obtained by taking the difference between XAS spectra with the photon helicity vector parallel (*μ*^+^) and antiparallel (*μ*^−^) to the applied magnetic field, respectively. The degree of circular polarization is 100% and for the sign convention, see ref.^20^. XMCD measurements were performed after first field-cooling to base temperature (~3K) in a 6T field. The sample was mounted at a grazing incidence angle of 30° with the applied magnetic field of ±6T along the x-ray beam direction. The XMCD results are obtained from an average over four *μ*^+^ and four *μ*^−^ scans of the photon energy across the absorption edges.

Element-specific measurements of the magnetization vs. field (*M*-*H*) and magnetization vs. temperature (*M*-*T*) were also carried out using synchrotron x-rays with photon energy tuned to the Cr *L*_3_ edge. For the *M*-*H* measurements, the field was swept at a constant velocity taking alternating on- and off-edge measurements on the fly at a constant temperature of 3K. Similarly, for the *M*-*T* measurements, on the fly on- and off-edge measurements were performed whilst ramping the temperature from 10K to 250K with a constant applied field of 20 mT. In both cases, the sample was initially field-cooled to 3K in a field of 6T. Measurements of the on-edge signal were normalized against the off-edge signal and the asymmetry was obtained between repeat measurements with both *μ*^+^ and *μ*^−^.
